# Non-significant results do not imply similarity or safety in NC-FET follicle-size comparisons: a clarification

**DOI:** 10.1093/hropen/hoag007

**Published:** 2026-01-30

**Authors:** Kentaro Iga

**Affiliations:** Division of Perinatology, Center for Fetal Diagnosis and Therapy, Seirei Hamamatsu General Hospital, Hamamatsu, Japan; Center for Medical Education, Graduate School of Medicine, Nagoya University, Nagoya, Japan

Dear Editor,

In a recent issue of *Human Reproduction Open*, Zhang *et al.* (2025) reported an important cohort study evaluating reproductive and perinatal outcomes in modified natural-cycle frozen embryo transfer (NC-FET) triggered at different follicle sizes. Given the scarce evidence guiding optimal timing of ovulation induction in NC-FET, their contribution is valuable. However, several aspects of the statistical interpretation require clarification, particularly the statements that outcomes were ‘similar’ and that triggering at smaller follicle sizes ‘was not associated with adverse outcomes’.

As previously noted elsewhere (Iga, 2026), many clinicians routinely—but incorrectly—interpret non-significant *P*-values as evidence of ‘no difference’. Yet, superiority testing, as used by Zhang *et al.* (2025), is designed to detect differences, not to confirm similarity or non-inferiority (Walker, 2019). When *P* values are non-significant, the only valid inference is that a difference was not detected; it does not follow that the groups are similar, equivalent, or clinically interchangeable.

To clarify what the data in Zhang *et al.* (2025) can support, I recalculated absolute risk differences (ARDs) for live birth rate (LBR) using Wilson intervals for single proportions and Newcombe’s method for between-group differences (Brown *et al.*, 2001, Fagerland *et al.*, 2015). The ≥18 mm reference group had an LBR of 39.0% (95% CI 37.8–40.2). ARDs (percentage points, 95% CI) were:

- <12 mm: −1.7% (−11.7 to +9.1)
- 12–12.9 mm: −2.6% (−9.0 to +4.1)
- 13–13.9 mm: −3.0% (−8.3 to +2.5)
- 14–14.9 mm: −3.3% (−7.5 to +1.0)
- 15–15.9 mm: −1.8% (−5.5 to +2.0)
- 16–16.9 mm: −1.4% (−4.6 to +1.9)
- 17–17.9 mm: −1.8% (−4.8 to +1.3)

Although none of the comparisons achieved statistical significance, several confidence intervals remain wide. Most notably, the <12 mm group is compatible with an 11.7-percentage-point lower LBR (27.3% vs 39.0% in the reference group). Such a magnitude of potential harm is clinically meaningful and cannot be dismissed.

To claim similarity or safety, one must first specify a clinically acceptable margin of reduced effectiveness (Δ). No such margin was prespecified by Zhang *et al.* (2025), and their study—retrospective in design—was not structured to evaluate non-inferiority. Without Δ, wide CIs cannot be interpreted in relation to clinical acceptability, and the conclusions of similarity or safety therefore exceed what the data can support (Walker, 2019).

Zhang *et al*. (2025) further conclude that triggering at smaller follicle sizes ‘was not associated with adverse outcomes’. In a multivariable superiority model, ‘not associated’ means only that an association was not detected; it is not evidence that harm is unlikely. Adjusted non-significant findings cannot compensate for imprecision due to wide ARDs or the absence of a non-inferiority framework.

This issue becomes particularly concerning in the patient-facing summary, which states that triggering below 18 mm ‘was not associated with negative reproductive and perinatal outcomes’. When the point estimate and 95% CI include the possibility of an 11.7-point lower LBR, communicating safety to patients is misleading.

Presenting ARDs and their CIs—as shown above—would enhance transparency by acknowledging the range of effects compatible with the data, including possible clinically relevant harm. This approach is increasingly encouraged in observational reproductive-medicine research as it reduces misinterpretation of non-significant results and expresses conclusions in clinically meaningful units.

Zhang *et al.* (2025) provide valuable descriptive data regarding NC-FET across a range of follicle sizes. My concerns relate not to the results themselves but to the interpretation placed upon them. A more cautious phrasing—emphasizing residual uncertainty and avoiding language implying similarity or safety—would improve the clarity and clinical applicability of this important work.
